# Reperfusion therapies in patients with acute ischaemic stroke and atrial fibrillation: data on safety and effectiveness from a multi-centre cohort study

**DOI:** 10.1007/s10072-024-07555-z

**Published:** 2024-05-22

**Authors:** Virginia Cancelloni, Mariachiara Buratti, Georgios Tsivgoulis, Karen L. Furie, Prasanna Tadi, Valeria Caso, Cecilia Becattini, Giancarlo Agnelli, Marialuisa Zedde, Azmil H. Abdul-Rahim, Andrea Alberti, Michele Venti, Ilaria Leone de Magistris, Monica Acciarresi, Cataldo D’Amore, Maria G. Mosconi, Ludovica A. Cimini, Manuel Cappellari, Jukka Putaala, Liisa Tomppo, Turgut Tatlisumak, Fabio Bandini, Simona Marcheselli, Alessandro Pezzini, Sung-I. I. Sohn, Gianni Lorenzini, Rossana Tassi, Francesca Guideri, Maurizio Acampa, George Ntaios, Efstathia Karagkiozi, George Athanasakis, Kostantinos Makaritsis, Dimitrios Sagris, Anastasia Adamou, Kostantinos Vadikolias, Lina Palaiodimou, Maria Chondrogianni, Nicola Mumoli, Franco Galati, Simona Sacco, Cindy Tiseo, Francesco Corea, Walter Ageno, Marta Bellesini, Giorgio Silvestrelli, Alfonso Ciccone, Michelangelo Mancuso, Giovanni Orlandi, Rosario Pascarella, Tiziana Tassinari, Christina Rueckert, Antonio Baldi, Danilo Toni, Federica Lettieri, Martina Giuntini, Enrico M. Lotti, Yuriy Flomin, Alessio Pieroni, Odysseas Kargiotis, Theodore Karapanayiotides, Panagiotis Halvatsiotis, Serena Monaco, Mario M. Baronello, Laszlò Csiba, Lilla Szabò, Alberto Chiti, Elisa Giorli, Massimo Del Sette, Davide Imberti, Dorjan Zabzuni, Boris Doronin, Vera Volodina, Patrik Michel, Peter Vanacker, Kristian Barlinn, Lars P. Pallesen, Jessica Kepplinger, Dirk Deleu, Vanessa Gourbali, Shadi Yaghi, Maurizio Paciaroni, RAF, R. A. F.-NOACs Investigators

**Affiliations:** 1 Stroke Unit and Division of Cardiovascular Medicine, University of Perugia, Perugia, Italy; 2 Second Department of Neurology, School of Medicine, National and Kapodistrian University of Athens, Attikon” University Hospital, Athens, Greece; 3 Division of Stroke and Cerebrovascular Diseases, Department of Neurology, The Warren Alpert Medical School of Brown University, Providence, RI USA; 4 Geisinger Medical Center, Danville, PA USA; 5 Neurology Unit, Stroke Unit, Arcispedale Santa Maria Nuova-IRCCS, Reggio Emilia, Italy; 6 Liverpool Centre for Cardiovascular Science at University of Liverpool, Liverpool John Moores University and Liverpool Heart & Chest Hospital, Liverpool, UK; 7 Cardiovascular and Metabolic Medicine, Institute of Life Course and Medical Sciences, University of Liverpool, Liverpool, UK; 8 Stroke Division, Mersey and West Lancashire Teaching Hospitals NHS Trust, St Helens, UK; 9 SC Neurologia, San Giovanni Battista Hospital, Foligno, Italy; 10 Stroke Unit, Ospedale Di Portogruaro, Portogruaro, Venice, Italy; 11 SSO Stroke Unit, UO Neurologia, DAI Di Neuroscienze, AOUI, Verona, Italy; 12 Department of Neurology, Helsinki University Central Hospital, Helsinki, Finland; 13 Deparment of Clinical Neurosciences, Institute of Neurosciences and Physiology, Sahlgrenska Academy at University of Gothenburg and Department of Neurology, Sahlgrenska University Hospital, Gothenburg, Sweden; 14 SC Neurologia, Villa Scassi Hospital, ASL 3 Genovese, Genoa, Italy; 15 Neurologia d’Urgenza E Stroke Unit, Istituto Clinico Humanitas, Rozzano, Milano Italy; 16 Department of Clinical and Experimental Sciences, Neurology Unit, University of Brescia, Brescia, Italy; 17 Deparment of Neurology, Keimyung University School of Medicine, Daegu, South Korea; 18 SC Di Medicina E Chirurgia d’Accettazione E d’Urgenza, Ospedali Di Pontedera E Volterra, Pontedera E Volterra, Italy; 19 Stroke Unit, AOU Senese, Siena, Italy; 20 Department of Internal Medicine, Faculty of Medicine, School of Health Sciences, University of Thessaly, Larissa, Greece; 21 Department of Neurology, Democritus University of Thrace, University Hospital of Alexandroupolis, Alexandroupolis, Greece; 22 UOC General Medicine, ASST Ovest Milanese, Legnano Hospital, Legnano, Italy; 23 Stroke Unit, Jazzolino Hospital, Vibo Valentia, Italy; 24 Department of Neurology, University of L’Aquila, Avezzano Hospital, Avezzano, Italy; 25 Department of Internal Medicine, Insubria University, Varese, Italy; 26 SC Di Neurologia E SS Di Stroke Unit, ASST Di Mantova, Mantua, Italy; 27 Clinica Neurologica, Azienda Ospedaliero-Universitaria, Pisa, Italy; 28 Neurologia, Ospedale Apuano, Massa Carrara, Italy; 29 Neuroradiology Unit, Azienda Unità Sanitaria Locale-IRCCS Di Reggio Emilia, Reggio Emilia, Italy; 30 Stroke Unit, Department of Neurology, Santa Corona Hospital, Pietra Ligure, Savona, Italy; 31 Neuroscience and Cognition, Utrecht University, Utrecht, Netherlands; 32 Department of Neurology and Psychiatry, Sapienza University of Rome, Rome, Italy; 33 UO Neurologia Presidio Ospedaliero Di Ravenna Azienda USL Della Romagna, Ravenna, Italy; 34 Stroke Unit and Neurorehabilitation Unit MC, Universal Clinic ‘Oberig’, Kiev, Ukraine; 35 UOSD Stroke Unit, Azienda Ospedale Università Padova, Padua, Italy; 36 Stroke Unit, Metropolitan Hospital, Piraeus, Greece; 37 2nd Department of Neurology, AHEPA University Hospital, Thessaloniki, Greece; 38 Second Department of Internal Medicine-Propaedeutic and Diabetes Center, Medical School, University General Hospital “Attikon”, National and Kapodistrian University of Athens, Athens, Greece; 39 Stroke Unit, Ospedale Civico, Palermo, Italy; 40 Stroke Unit, University of Debrecen, Debrecen, Hungary; 41 Stroke Unit, Department of Neurology, Sant’Andrea Hospital, La Spezia, Italy; 42 UOC Neurologia, IRCCS Ospedale Policlinico San Martino, Genoa, Italy; 43 Department of Internal Medicine, Ospedale Civile Di Piacenza, Piacenza, Italy; 44 Municipal Budgetary Healthcare Institution of Novosibirsk, Novosibirsk State Medical University, City Clinical Hospital N°1, Novosibirsk, Russia; 45 Centre Cérébrovasculaire, Service de Neurologie, Département des Neurosciences Cliniques Centre Hospitalier Universitaire Vaudois, Lausanne, Switzerland; 46 Department of Neurology, Born Bunge Institute, Antwerp University Hospital, Antwerp, Belgium; 47 Department of Neurology, Dresden University Stroke Center, Dresden, Germany; 48 Hamad Medical Corporation, Doha, Qatar; 49 Department of Neurology, Evangelismo Hospital, Athens, Greece

**Keywords:** ischaemic stroke, atrial fibrillation, reperfusion therapies, intravenous thrombolysis, endovascular therapy, outcome

## Abstract

**Background:**

Intravenous thrombolysis (IVT) and/or endovascular therapy (EVT) are currently considered best practices in acute stroke patients. Data regarding the efficacy and safety of reperfusion therapies in patients with atrial fibrillation (AF) are conflicting as regards haemorrhagic transformation, mortality, and functional outcome. This study sought to investigate for any differences, in terms of safety and effectiveness, between AF patients with acute ischaemic stroke (AIS) treated and untreated with reperfusion therapies.

**Methods:**

Data from two multicenter cohort studies (RAF and RAF-NOACs) on consecutive patients with AF and AIS were analyzed to compare patients treated and not treated with reperfusion therapies (IVT and/or EVT). Multivariable logistic regression analysis was performed to identify independent predictors for outcome events: 90-day good functional outcome and mortality. A propensity score matching (PSM) analysis compared treated and untreated patients.

**Results:**

Overall, 441 (25.4%) were included in the reperfusion-treated group and 1,295 (74.6%) in the untreated group. The multivariable model suggested that reperfusion therapies were significantly associated with good functional outcome. Rates of mortality and disability were higher in patients not treated, especially in the case of higher NIHSS scores. In the PSM comparison, 173/250 patients (69.2%) who had received reperfusion therapies had good functional outcome at 90 days, compared to 146/250 (58.4%) untreated patients (p = 0.009, OR: 1.60, 95% CI:1.11–2.31).

**Conclusions:**

Patients with AF and AIS treated with reperfusion therapies had a significantly higher rate of good functional outcome and lower rates of mortality compared to those patients with AF and AIS who had undergone conservative treatment.

**Supplementary Information:**

The online version contains supplementary material available at 10.1007/s10072-024-07555-z.

## Introduction

Atrial fibrillation (AF) is the most prevalent sustained cardiac arrhythmia encountered in clinical practice, present in approximately 15% to 20% of all ischaemic strokes [1]. Moreover, AF has been reported to increase the risk of ischemic stroke by 4 to fivefold, especially in patients over 80 years [2, 3]. Ischaemic stroke patients with concomitant AF generally have higher baseline stroke severity with larger severe hypoperfusion volumes and larger infarct size, and more frequent severe haemorrhagic transformation [4, 5]. Moreover, patients with AF are more often older and therefore carry a higher vascular risk due to the presence of vascular comorbidities which are known to increase the risks of both AF and AIS; this is, despite the use of oral anticoagulants at the time of stroke onset [6, 7]. Because of this, patients with acute ischaemic stroke (AIS) and AF more frequently have more severe ischaemic strokes with poorer outcomes, greater disability and higher mortality rates, when compared to AIS patients without a history of AF [8].

Acute reperfusion therapies, that are intravenous thrombolysis (IVT) and/or endovascular therapy (EVT), are currently considered best practices for the management of AIS, as their administration is associated with better functional outcomes, along with lower morbidity, mortality, and long-term disability rates, compared to patients not treated [9–12]. However, data regarding the efficacy and safety of reperfusion therapies in patients with AF have been conflicting concerning functional outcome, as well as the rates of mortality, morbidity and haemorrhagic transformation.

A 2021 metanalysis [13] including a total of 8,509 patients compared the outcomes associated with the administration of IVT treatment in AIS patients with AF versus non-AF patients and reported that the AF cohort, overall, had significantly lower rates of good functional outcomes (mRS 0–2), as well as significantly higher mortality. Besides the impact on functional outcome, AF was suggested to be an independent risk factor correlated with non-early recanalization [14]. Another study [15] reported opposite results, with patients with AF and AIS treated with IVT showing more frequently excellent outcomes (mRS 0–1) and good outcomes (mRS 0–2) at 3 months and less frequent death when compared with non-treated patients.

Likewise, data regarding the effect of endovascular therapy (EVT) in ischaemic stroke patients and AF has been conflicting. A recent metanalysis [16] including 6,543 patients reported significantly lower rates of mRS 0–2 among patients with AIS and AF treated with EVT, when compared to patients without AF, as well as higher mortality. In contrast, similar rates of 90-day good functional outcome were reported for patients with and without AF and large vessel occlusion treated with EVT [17].

To the best of our knowledge, no randomized clinical trial has been designed to investigate for a possible interaction between AF and reperfusion therapies and any effect on functional outcome and mortality. Presently, it remains unclear whether ischaemic stroke patients with AF might respond differently to reperfusion therapies and whether the presence of AF might have any consequences on stroke outcomes.

In light of the above, the aim of our study was to investigate for any effects associated with the use of reperfusion therapies in patients with AIS and AF; specifically, 90-day functional outcome and mortality.

## Methods

### Study design and population

We performed a post-hoc analysis of pooled individual data from a previously published international collaboration of investigator-initiated prospective cohort studies; namely the RAF and RAF-NOACs studies. Details regarding the study designs and methods can be obtained from the published studies [18, 19].

The studies included patients with AIS who completed a systematic 3-month follow-up after the index event and who had a diagnosis of nonvalvular AF, either known before or post-index event. The RAF study included patients treated with either vitamin K antagonists (VKAs) or direct oral anticoagulants (DOACs); the RAF-NOACs study included only patients treated with DOACs.

Exclusion criteria of both studies were: (1) intracerebral haemorrhage that had occurred before the index event; (2) mechanical heart valves; (3) rheumatic or severe mitral valve stenosis; (4) long-term secondary stroke prevention with antiplatelets only; or (5) missing information on antithrombotic treatment before and after the index event.

Standard stroke unit care, monitoring, and treatment were provided according to current international recommendations for AIS.

### Data collection

Data were collected as described in prior publications [18, 19]: local investigators were responsible for compiling standardised forms with predefined variables using individual patient data from corresponding study databases. Completed forms were sent to the coordinating centre in Perugia, Italy, where the pooled analysis was performed. The corresponding author and all of the co-authors had full access to the data and took responsibility for the integrity of the analysis.

### Baseline data

The following baseline variables were collected: age, sex, and information on any ongoing anticoagulation treatment with VKAs or DOACs at both admission and after the index event. DOAC therapy was defined as the following: apixaban 2.5 mg or 5 mg twice daily; dabigatran 110 mg or 150 mg twice daily; edoxaban 30 mg or 60 mg once daily; or rivaroxaban 15 mg or 20 mg once daily. Treatment with VKAs was defined as acenocoumarol/warfarin. The choice of treatment was decided by the treating physician. Information about NIHSS scores, blood glucose levels, and systolic blood pressure values on admission were also recorded for all the patients. The following risk factors were collected: history of hypertension (blood pressure of ≥ 140/90 mmHg at least twice before stroke or antihypertensive therapy), current smoking, hyperlipidaemia (total cholesterol ≥ 200 mg/dL or triglyceride ≥ 140 mg/dL or lipid-lowering therapy), history of symptomatic ischaemic heart disease (myocardial infarction, angina, existence of multiple lesions on thallium heart isotope scan or evidence of coronary disease on coronary angiography), history of congestive heart failure, current alcohol abuse (≥ 300 g per week), and history of previous AIS or transient ischaemic attack (TIA). Moreover, information regarding any administered reperfusion therapies was also obtained. Acute reperfusion therapies were defined as IVT with r-tPA at the dosage of 0.9 mg/Kg and EVT that were delivered as per standard local protocol. On admission, either a cerebral CT without contrast or an MRI scan was performed on all patients to exclude intracranial haemorrhage. A second brain CT scan or MRI was also performed 24 to 72 h from stroke onset on all patients. Haemorrhagic transformation (HT) was defined on CT as any degree of hyperdensity within the area of low attenuation and was classified as either hemorrhagic infarction or parenchymal hematoma. On MRI, HT was defined as hypointensity on axial T1- and T2-weighted images. HT was considered symptomatic if accompanied by a decline in neurological status (an increase of ≥ 4 points in NIHSS score) or death, in the absence of any bleeding evidence on the first CT.

### Evaluation of outcome

Follow-up was initiated at the time of the index event and lasted 3 months. Follow-up visits and outcome adjudication were performed by local investigators, in a non-blinded manner.

Primary outcome measures were death or disability and good functional outcome at 90 days. Disability was defined as a modified Rankin Scale (mRS) of 3–5. Good functional outcome was defined as a mRS of 0–2.

Secondary outcome was defined as mortality at 90 days.

### Statistical analysis

We compared baseline characteristics of reperfusion-treated and not-treated patients using the χ2 test for categorical variables or the Mann–Whitney *U* test for continuous variables. Patient characteristics are summarized for continuous variables as mean ± SD if they were normally distributed and as median and interquartile range if they were not normally distributed. While categorical variables were presented as absolute numbers and percentages. The risk of the study outcomes between reperfusion-treated and untreated cohorts was compared using logistic regression analysis. Results were reported as odds ratios (OR) and 95% Confidence Intervals (CIs). Multivariable analysis was performed using logistic regression to investigate for any independent predictors of the primary outcomes: good functional outcome (mRS 0–2) and mortality assessed at 90 days from AIS. The independent variables included in the multivariable models were: age, male sex, NIHSS score, systolic blood pressure (SBP), blood glucose levels at admission, history of diabetes, history of hypertension, hyperlipidemia, presence of paroxysmal AF, history of stroke or TIA, current smoking habit, current alcohol use, history of congestive heart failure (CHF), history of myocardial infarction (MI), oral anticoagulants (DOACs or warfarin) at admission.

Using propensity score matching (PSM), a further analysis was performed that compared patients treated and not treated with reperfusion therapies. This score was calculated by including selected variables from the univariate analysis, using backward stepwise analysis with a 0.1 level as a screening criterion. Subsequently, matching was carried out with a 1:1 ratio across the groups, without replacement, and with a forced preservation of bridging cases. Data were analyzed using the SPSS/PC Win package 25.0. Statistical significance was defined as *p* < *0.05*.

## Results

### Characteristics of the study population

The overall cohort for this analysis included a total of 1,736 patients with AIS and known or newly diagnosed AF: 441 patients (25.4%) were treated with reperfusion therapies (IVT and/or EVT), while 1,295 (74.6%) received conservative treatment. Of the treated patients, 346 (78.4%) received IVT, 51 (11.6%) received IVT plus EVT, and 44 (10%) received only EVT. We combined all the patients who had received IVT and/or EVT in one group and considered them as the treated group.

Patients treated with reperfusion therapies were on average younger (73.5 years vs. 76.8 years, p < 0.001) and had more severe strokes according to NIHSS score (11.7 vs 7.2, p < 0.001). The two groups differed significantly for their systolic blood pressure values (153 mmHg vs. 149.4 mmHg, p = 0.009), known or newly diagnosed paroxysmal AF (48% vs 39.9%, p = 0.004), and history of stroke or TIA (18.8% vs. 28.7%, p < 0.001). Patients who received reperfusion therapies less frequently received anticoagulation therapies on admission (15.4% vs 28.5%, p < 0.001) and were more likely to have been treated with oral anticoagulants after the index event (86.8% vs. 76.4%, p < 0.001). The baseline characteristics of the patients are summarized in Table S1 in the Supplemental Materials.

### Rates and predictive factors of outcome events

At 90 days, 668 patients (38.5%) were deceased or disabled (mRS 3–6) and 1,033 (59.5%) had good functional outcome (mRS 0–2).

The results from the univariate analysis suggested that those patients who were deceased or disabled were older, more frequently males, had more severe ischaemic stroke (according to NIHSS score), more often had paroxysmal AF, hyperlipidemia and higher blood glucose levels, and were more likely to be current smokers and alcohol abusers. Rates of patients treated with reperfusion therapies did not differ significantly in the two groups (23,9% vs 26,1%, p = 0.3). Primary outcomes at 90 days are summarized in Table S2 in the Supplemental Materials. Regarding mortality (Table S3 in Supplemental Materials), the univariate analysis indicated that older age, higher NIHSS score, and higher blood glucose levels were more frequent in patients who were deceased at 90 days. Death at 90 days was less frequent in patients treated with reperfusion therapies (4.5% vs 7.9%, p = 0.006). No difference was observed in terms of good functional outcome (Table 1).

**Table 1:** Univariate analysis on good functional outcome and death at 90 days in patients treated and not treated

	IVT, EVT or combined [n. 441]	No treatment [n. 1295]	*P value*
Good functional outcome	270 (61.2%)	762 (58.8%)	0.3
Death at 90 days	18 (4.5%)	102 (7.9%)	0.006

Good functional outcome is defined as a modified Rankin Scale of 0–2

*IVT* intravenous thrombolysis, *EVT* endovascular therapy

No statistically significant difference was found between the two groups regarding the rates of haemorrhagic transformation (14.2% vs 12.1%, p = 0.2), even though the incidence of haemorrhagic transformation was higher in patients treated with reperfusion therapies.

The multivariable model suggested that older age (OR:0.97, 95% IC:0.96–0.99) and higher NIHSS score (OR:0.84, 95% IC:0.81–0.86) were inversely correlated with good functional outcome. Treatment with oral anticoagulants after the index event (OR:4.5, 95% IC:3.26–6.17) resulted being an independent predictive factor for good functional outcome (Table 2). Regarding secondary outcome, independent predictive factors of mortality were age (OR:1.04, 95% IC:1.01–1.07), male sex (OR:1.15, 95% IC:0.70–1.89), NIHSS score (OR:1.09, 95% IC:1.06–1.12), and oral anticoagulation after the index event (OR:0.14, 95% IC:0.08–0.22) (Table 3). Results from the multivariable model suggested that reperfusion therapies (IVT and/or EVT) were significantly associated with a good functional outcome, but not with lower mortality.

**Table 2:** Multivariate model to predict good functional outcome (mRS 0–2) in patients with AF and acute ischemic stroke

	O.R. [95% CI]	*P value*
Revascularization (IVT + EVT)	2.28 [1.65 – 3.17]	< 0.001
Age	0.97 [0.96 – 0.99]	0.001
Male sex	1.20 [0.90 – 1.57]	0.20
NIHSS	0.84 [0.81 – 0.86]	< 0.001
SBP	0.99 [0.99 – 1.00]	0.31
Blood glucose levels	0.99 [0.99 – 1.00]	0.02
Diabetes	1.09 [0.78 – 1.52]	0.59
Hypertension	1.06 [0.76 – 1.49]	0.69
Hyperlipidemia	1.36 [1.02 – 1.80]	0.03
Paroxysmal AF	0.97 [0.75 – 1.27]	0.87
History of stroke or TIA	0.86 [0.64 – 1.16]	0.34
Current smoker	1.17 [0.86 – 1.58]	0.30
Alcoholism	1.10 [0.63 – 1.89]	0.72
CHF	0.76 [0.54 – 1.07]	0.12
History of MI	0.98 [0.67 – 1.43]	0.93
OAT after the index event	4.50 [3.26 – 6.17]	< 0.001

*IVT* intravenous thrombolysis, *EVT* endovascular therapy, *NIHSS* National Institute of Health stroke scale, *SBP* systolic blood pressure, *AF* atrial fibrillation, *TIA* transient ischemic attack, *CHF* congestive heart failure, *MI* myocardial infarction, *OAT* oral anticoagulation therapy. *CI* confidence interval, *OR* odds ratio

**Table 3:** Multivariate model to predict mortality in patients with AF and acute ischemic stroke

	O.R. [95% CI]	*P value*
Revascularization (IVT + EVT)	0.61 [0.33 – 1.11]	0.10
Age	1.04 [1.01- 1.07]	0.001
Male sex	1.15 [0.70 – 1.89]	0.001
NIHSS	1.09 [1.06 – 1.12]	< 0.001
SBP	1.00 [0.99 – 1.01]	0.50
Blood glucose levels	1.00 [0.99 – 1.00]	0.09
Diabetes	1.02 [0.59 – 1.76]	0.93
Hypertension	0.91 [0.50 – 1.65]	0.76
Hyperlipidemia	0.89 [0.54 – 1.48]	0.67
Paroxysmal AF	1.16 [0.72 – 1.85]	0.53
History of stroke or TIA	0.69 [0.41 – 1.16]	0.16
Current smoker	1.44 [0.85 – 2.41]	0.16
Alcoholism	1.23 [0.49 – 3.06]	0.64
CHF	1.17 [0.66 – 2.06]	0.57
History of MI	1.23 [0.68 – 2.24]	0.48
OAT after the index event	0.14 [0.08 – 0.22]	< 0.001

*IVT* intravenous thrombolysis, *EVT* endovascular therapy, *NIHSS* National Institute of Health stroke scale, *SBP* systolic blood pressure, *AF* atrial fibrillation, *TIA* transient ischemic attack, *CHF* congestive heart failure, *MI* myocardial infarction, *OAT* oral anticoagulation therapy, *CI* confidence interval, *OR* odds ratio

Regarding the severity of the index event, rates of mortality and disability were found to be higher in patients not treated with reperfusion therapies, especially in those with higher NIHSS scores (Table S4 in Supplemental Materials, Fig. 1).

**Fig. 1:**
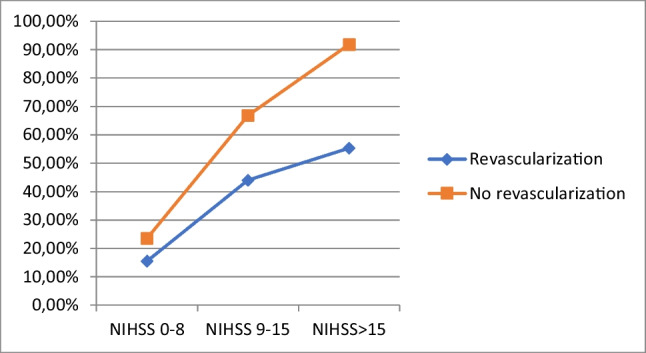
Mortality and disability rates in patients treated and not treated with IVT and/or EVT, divided by stroke severity. NIHSS National Institute of Health Stroke Scale

Of the treated patients, 346 (78.4%) received IVT, 44 (10%) received EVT only, and 51 (11.6%) received IVT plus EVT. Rates of death and worse outcomes (death or disability) at 90 days based on the type of acute treatment administered are reported in Table 4. When compared to untreated patients, patients treated with IVT only, EVT only or IVT plus EVT had lower rates of death at 90 days (adjusted OR:0.57, 95% CI:0.33–0.97 for IVT and adjusted OR:0.56, 95% CI:0.13–2.33 for EVT; OR for IVT + EVT not applicable) as well as lower rates of death and disability combined (OR:0.71, 95% CI:0.55–0.91 for IVT, OR:1.31, 95% CI:0.72–2.38 for EVT, and OR:1.17, 95% CI:0.67–2.06 for IVT plus EVT). Because of the small number of patients in EVT and IVT + EVT groups, adjusted OR could only be performed for the IVT cohort.

**Table 4:** Rates of death and worse outcomes at 90 days based on the type of treatment administered

	IVT only [n. 346]	EVT only [n. 44]	IVT + EVT [51]	No treatment [n.1295]
Death	16 (4.6%)	2 (4.5%)	0 (0%)	102 (7.9%)
Death or disability	115 (33.2%)	21 (47.7%)	23 (45.1%)	533 (41.2%)

Disability is defined as a modified Rankin Scale of 3–5

*IVT* intravenous thrombolysis, *EVT* endovascular therapy

Following PSM, 250 patients who had received reperfusion therapies were compared with 250 who had not (Table 5). No significant differences between the two groups were observed. As for the 90-day functional outcome, 173/250 patients (69.2%) who had received reperfusion therapies, had a good functional outcome, compared to 146/250 (58.4%) patients who had undergone conservative treatment (p = 0.009, OR:1.60, 95% IC:1.11–2.31).

**Table 5:** Univariate comparison between patients treated and not treated with reperfusion therapies after propensity score matching

	Treated with IVT or EVT or combined [n. 250]	No revascularization [n. 250]	*P* value
Age (mean,years)	75.2 ± 9.4	74.6 ± 10.4	0.21
Sex (M)	123 (49.2%)	116 (46.4%)	0.59
NIHSS score	9.8 ± 5.4	9.1 ± 7.2	0.23
Blood glucose levels	126.8 ± 42.4	123.7 ± 44.1	0.42
SBP	151.2 ± 23.8	153.6 ± 24.2	0.28
Diabetes	46 (18.4%)	46 (18.4%)	1.00
Hypertension	197 (78.8%)	189 (75.6%)	0.46
Hyperlipidemia	83 (33.2%)	79 (31.6%)	0.77
Paroxysmal AF	104 (41.6%)	113 (45.2%)	0.47
History of stroke or TIA	64 (25.6%)	61 (24.4%)	0.84
Current smoker	69 (27.6%)	75 (30%)	0.62
Alcoholism	18 (7.2%)	14 (5.6%)	0.58
CHF	39 (15.6%)	46 (18.4%)	0.48
History of MI	33 (13.2%)	29 (11.6%)	0.68
OAT on admission	46 (18.4%)	47 (18.8%)	1.00

*IVT* intravenous thrombolysis, *EVT* endovascular therapy, *NIHSS* National Institute of Health stroke scale, *SBP* systolic blood pressure, *AF* atrial fibrillation, *TIA* transient ischemic attack, *CHF* congestive heart failure, *MI* myocardial infarction, *OAT* oral anticoagulation therapy

## Discussion

In this international, multicenter, prospective cohort study, we found that patients with known or newly diagnosed AF and AIS treated with reperfusion therapies (IVT and/or EVT) had statistically significant higher rates of good functional outcomes (mRS 0–2), compared to patients not receiving reperfusion therapies.

Our results are in line with previous studies [15, 20]. Specifically, Padjen et al. [15] reported that patients with AF and AIS who had received IVT were more likely to have excellent outcomes (mRS 0–1) and good outcomes (mRS 0–2) at 3 months, when compared with not treated patients (excellent outcome 58.8% vs 23.8%, OR:4.59, 95% CI:2.00–10.52; good outcome 64.7% vs 27.8%, OR:4.75, 95% CI:2.07–10.92). The same study reported that the delivery of IVT was associated with significantly lower rates of mortality (OR:0.19, 95% CI:0.05–0.77, p = 0.021), which was not confirmed in our cohort analysis. This may be explained by the fact that patients with AF not treated with reperfusion therapies had higher median NIHSS values (14) compared to patients included in our analysis.

Regarding EVT, our results are not in agreement with those of previous studies. That is, a recent metanalysis [16] reported significantly lower rates of good functional outcome among patients with AIS and AF treated with EVT, when compared to AIS patients without AF (OR:0.65, 95%CI:0.52–0.81, p < 001), as well as higher mortality (OR:1.47, 95%CI:1.12–1.92, p = 0.005). Our results suggest that, despite the type of administered treatment (IVT only, EVT only or IVT plus EVT), patients treated with reperfusion therapies had lower rates of both death at 90 days and death and disability combined. However, because of the small number of patients in EVT and IVT + EVT groups, adjusted OR could only be carried out for the IVT treatment cohort.

In line with previous study findings, the multivariate model indicated that age, NIHSS score and oral anticoagulation after the index event were predictive factors for both good functional outcome and mortality.

Results from the univariate analysis differed from the multivariate model both in terms of good functional outcome and mortality rates. In our opinion, this might be explained by the heterogeneity of patient characteristics and by the fact that the two groups were mismatched for several risk factors. In particular, patients treated with IVT, EVT or both, had higher NIHSS values (11.7 vs 7.2, p < 0.001) compared to those not treated, and consequently, had more severe ischaemic strokes; despite the median younger age. Moreover, in the group of non-treated patients, there was a higher number of patients who had had a previous stroke or TIA.

Additionally, most of the patients did not receive any reperfusion therapy (1,295/1,736 patients). We hypothesise that this was due to the fact that the cohorts included AF patients on anticoagulation treatment. Moreover, our study relied upon data from RAF and RAF-NOACs studies, which have been carried out before the availability of both EVT and emergency DOACs dosing for patients with AF. This might have excluded several patients from being candidates for reperfusion therapies, especially anticoagulated patients who would have undergone EVT. In line with previous findings, all the patients treated with any type of reperfusion therapy (IVT only, EVT only and IVT + EVT) had lower rates of both death and worse outcomes (death plus disability). No patients treated with either IVT or EVT were recorded as deceased at 90-day follow-up.

Because of the heterogeneity of our patient cohorts, we performed an adjunctive analysis using PSM, comparing 250 patients who had received revascularization therapies and 250 patients not treated patients. From PSM, no statistically significant differences were found between the two groups of patients. However, patients who received reperfusion therapies had a significantly higher incidence of good functional outcome at 90 days.

Our study had some limitations. First, it was a post-hoc analysis of observational, non-randomized studies and consequently, a selection bias of the studied populations could not be excluded; despite the adopted PSM approach. Second, the cohort of patients who received reperfusion therapies (and in particular EVT and IVT + EVT) was small, so adjusted OR could not be calculated. Moreover, data regarding mRS pre-event were not available for all the patients. Based on current guidelines, patients who received reperfusion therapies had an mRS pre-event of 0–2. This variable was not available for the untreated patients who, plausibly, might have had higher pre-event mRS. For this reason, we could not perform a comparison between mRS before and after the index event. It cannot be ruled out that this may have created a bias in our results. Finally, information regarding the time elapsed from symptom onset to treatment initiation was not available. Certainly, this variable would have been important to consider as regards the study outcomes. However, all patients treated with reperfusion therapies presented with symptom onset within the therapeutic window according to the current guidelines.

A strength of the study was that it reflected real-life experiences. Moreover, despite being retrospective, patients were collected prospectively and in a multicentered modality, allowing for a large cohort.

In conclusion, our results suggest that patients with AF and AIS could obtain benefits from treatment with intravenous thrombolysis, mechanical thrombectomy or a combination of both.

In this particular population with more severe baseline ischaemic stroke, poorer outcomes and greater morbidity and mortality, all treatments appear to be safe and correlate to a few rates of death and disability as well as higher rates of good functional outcome at 90 days.

## Supplementary Information

Below is the link to the electronic supplementary material.

Supplementary file1 (DOCX 26 KB)
