# Supplementary material for: Reperfusion therapies in patients with acute ischaemic stroke and atrial fibrillation: data on safety and effectiveness from a multi-centre cohort study
Source: Neurol Sci. 2024 May 22;45(10):4895–902. doi: 10.1007/s10072-024-07555-z (PMC11422471; doi:10.1007/s10072-024-07555-z)
Supplement: Supplementary file 1 — Supplementary file1 (DOCX 26 KB) [file 10072_2024_7555_MOESM1_ESM.docx]

|  | **Treated with IVT or EVT or combined [n. 441]** | **No revascularization**  **[n. 1295]** | ***P value*** |
| --- | --- | --- | --- |
| Age | 73.5±9.9 | 76.8±9.7 | <0.001 |
| NIHSS | 11.7±6.2 | 7.2±6.7 | <0.001 |
| Blood glucose levels | 124.8±39.5 | 128.3±46.7 | 0.11 |
| SBP | 153.0±24.8 | 149.4±24.6 | 0.009 |
| Male sex | 212 (48.0%) | 597 (46.1%) | 0.42 |
| Paroxysmal AF | 212 (48.0%) | 517 (39.9%) | 0.004 |
| Hypertension | 328 (74.3%) | 1013 (78.2%) | 0.07 |
| History of stroke or TIA | 83 (18.8%) | 372 (28.7%) | <0.001 |
| Current smoker | 125 (28.3%) | 409 (31.5%) | 0.21 |
| Alcoholism | 28 (6.3%) | 93 (7.1%) | 0.63 |
| CHF | 70 (15.8%) | 240 (18.5%) | 0.20 |
| History of MI | 55 (12.4%) | 200 (15.4%) | 0.11 |
| Hyperlipidemia | 139 (31.5%) | 436 (33.6%) | 0.41 |
| OAT on admission | 68 (15.4%) | 371 (28.5%) | <0.001 |
| OAT after the index event | 383 (86.8%) | 990 (76.4%) | <0.001 |

**Table S1.** Demographic and clinical characteristics of patients at baseline.

IVT intravenous thrombolysis; EVT endovascular therapy; NIHSS National Institute of Health stroke scale; SBP systolic blood pressure; AF atrial fibrillation; TIA transient ischemic attack; CHF congestive heart failure; MI myocardial infarction; OAT oral anticoagulation therapy.

|  | **Death or disability**  **[n. 668]** | **Good functional outcome [n. 1033]** | ***P value*** |
| --- | --- | --- | --- |
| Age | 78.7±9.1 | 74.1±9.8 | <0.001 |
| NIHSS | 12.4±7.3 | 5.7±5.1 | <0.001 |
| Blood glucose levels | 133.9±50.0 | 123.2±41.1 | <0.001 |
| SBP | 152.5±25.9 | 149.1±23.8 | 0.005 |
| Male sex | 259 (38.7%) | 532 (51.5%) | <0.001 |
| Paroxysmal AF | 244 (36.5%) | 474 (45.8%) | <0.001 |
| History of stroke or TIA | 187 (27.9%) | 258 (24.9%) | 0.16 |
| Current smoker | 165 (24.7%) | 360 (34.8%) | <0.001 |
| Alcoholism | 35 (5.2%) | 83 (8.0%) | 0.03 |
| CHF | 131 (19.6%) | 171 (16.5%) | 0.10 |
| History of MI | 102 (15.2%) | 145 (14.2%) | 0.48 |
| Hyperlipidemia | 193 (28.8%) | 372 (36.1%) | 0.003 |
| OAT after the index event | 162 (24.2%) | 267 (25.8%) | 0.53 |
| Revascularization (IVT+EVT) | 160 (23.9%) | 270 (26.1 %) | 0.32 |

**Table S2.** Primary outcomes at 90 days.

Disability outcome is defined as a modified Rankin scale of 3-5. Good functional outcome is defined as a modified Rankin Scale of 0-2.

NIHSS National Institute of Health stroke scale; SBP systolic blood pressure; AF atrial fibrillation; TIA transient ischemic attack; CHF congestive heart failure; MI myocardial infarction; OAT oral anticoagulation therapy; IVT intravenous thrombolysis; EVT endovascular therapy.

|  | **Dead [n. 120]** | **Not dead [n. 1581]** | ***P value*** |
| --- | --- | --- | --- |
| Age | 81.3±9.4 | 75.5±9.7 | <0.001 |
| NIHSS | 14.6±8.2 | 7.9±6.5 | <0.001 |
| Blood glucose levels | 141.9±53.7 | 126.4±44.2 | <0.001 |
| SBP | 149.3±25.8 | 150.5±24.7 | 0.60 |
| Male sex | 52 (43.3%) | 739 (46.7%) | 0.51 |
| Paroxysmal AF | 43 (35.8%) | 675 (42.6%) | 0.15 |
| History of stroke or TIA | 31 (25.8%) | 414 (26.1%) | 1.00 |
| Current smoker | 37 (30.8%) | 488 (30.8%) | 1.00 |
| Alcoholism | 10 (8.3%) | 108 (6.8%) | 0.46 |
| CHF | 25 (20.8%) | 277 (17.5%) | 0.39 |
| History of MI | 24 (20.4%) | 233 (14.7%) | 0.08 |
| Hyperlipidemia | 36 (30.1%) | 529 (33.4%) | 0.48 |
| OAT after the index event | 36 (30.2%) | 393 (24.8%) | 0.23 |
| Revascularization (IVT+EVT) | 18 (15.3%) | 412 (26.4%) | 0.006 |

**Table S3.** Secondary outcome at 90 days.

NIHSS National Institute of Health stroke scale; SBP systolic blood pressure; AF atrial fibrillation; TIA transient ischemic attack; CHF congestive heart failure; MI myocardial infarction; OAT oral anticoagulation therapy; IVT intravenous thrombolysis; EVT endovascular therapy.

|  | **NIHSS 0-8 [148]** | **NIHSS 9-15 [166]** | **NIHSS>15 [114]** |
| --- | --- | --- | --- |
| Revascularization [430] | 23 [15.5%] | 73 [44.0%] | 63 [55.3%] |
| No revascularization | 205 [23.5%] | 141 [66.8%] | 156 [91.8%] |

**Table S4.** Mortality and disability rates in patients treated and not treated with IVT and/or EVT, divided by stroke severity.

NIHSS National Institute of Health Stroke Scale.
